# Vocalizations of wild West Indian manatee vary across subspecies and geographic location

**DOI:** 10.1038/s41598-023-37882-8

**Published:** 2023-07-07

**Authors:** Jessica D. Reyes-Arias, Beth Brady, Eric A. Ramos, Yann Henaut, Delma Nataly Castelblanco-Martínez, Maria Maust-Mohl, Linda Searle, Gabriela Pérez-Lachaud, Héctor M. Guzmán, Héctor Poveda, Fernando Merchan, Kenji Contreras, Javier E. Sanchez-Galan, Kristi A. Collom, Marcelo O. Magnasco

**Affiliations:** 1 Conservación de la Biodiversidad, El Colegio de la Frontera Sur, 77014 Chetumal, Quintana Roo Mexico; 2 Mote Marine Laboratory, 1600 Ken Thompson Parkway, Sarasota, FL 34236 USA; 3 Fundación Internacional para la Naturaleza y la Sustentabilidad (FINS), 77014 Chetumal, Quintana Roo Mexico; 4 The Rockefeller University, 1230 York Ave., New York, NY 10065 USA; 5 Universidad Autónoma del Estado de Quintana Roo, 77039 Chetumal, Quintana Roo Mexico; 6 Consejo Nacional de Ciencia y Tecnología, 03940 Ciudad de México, Mexico; 7 Department of Psychology, Manhattan College, Riverdale, New York, NY 10471 USA; 8 ECOMAR, PO Box 1234, Belize City, Belize; 9 Smithsonian Tropical Research Institute, 0843-03092 Panama City, Panama; 10 Grupo de Investigación en Sistemas de Comunicaciones Digitales Avanzados (GISCDA), Facultad de Ingeniería de Eléctrica, Universidad Tecnológica de Panamá, El Dorado, Panama City, 0819-07289 Panama; 11 Facultad de Ingeniería de Sistemas Computacionales, Universidad Tecnologica de Panama, Campus Victor Levi Sasso, Panama, Panama; 12 Department of Psychology, Hunter College, City University of New York, New York, NY 10065 USA

**Keywords:** Computational biology and bioinformatics, Ecology, Zoology

## Abstract

Geographic variation in the vocal behavior of manatees has been reported but is largely unexplored. Vocalizations of wild West Indian manatees (*Trichechus manatus*) were recorded with hydrophones in Florida from Florida manatees (*Trichechus manatus latirostris*), and in Belize and Panama from Antillean manatees (*Trichechus manatus manatus*) to determine if calls varied between subspecies and geographic regions. Calls were visually classified into five categories: squeaks, high squeaks, squeals, squeak-squeals, and chirps. From these five categories, only three call types (squeaks, high squeaks and squeals) were observed in all three populations. Six parameters from the temporal and frequency domains were measured from the fundamental frequency of 2878 manatee vocalizations. A repeated measures PERMANOVA found significant differences for squeaks and high squeaks between each geographic location and for squeals between Belize and Florida. Almost all measured frequency and temporal parameters of manatee vocalizations differed between and within subspecies. Variables that may have influenced the variation observed may be related to sex, body size, habitat and/or other factors. Our findings provide critical information of manatee calls for wildlife monitoring and highlight the need for further study of the vocal behavior of manatees throughout their range.

## Introduction

Geographic separation can result in acoustic differences in animal vocalizations. Macrogeographic variation is observed between isolated populations, whereas microgeographic variations are associated with closely neighboring species that have the potential to interbreed^1^. Intraspecific variation in vocalizations in animals may occur due to factors such as genetic differences (birds^2^); properties of the habitat^3^; and species dispersion (birds^4,5^) among others. There have been many studies on geographic variation in multiple marine mammal species including harbor seals (*Phoca vitulina*)^6^, bearded seals (*Erignathus barbatus*)^7^, blue whales (*Balaenoptera musculus*)^8^, and bottlenose dolphins (*Tursiops truncatus*)^9^. Comparative studies of geographic differences in vocalizations and their acoustic parameters within species can improve our understanding of the evolution of the vocal repertoire and factors that influence the variation in vocalizations. While our understanding of the acoustic characteristics of West Indian manatee vocalizations has increased^10–13^, how the vocalizations vary by region or subspecies is still poorly understood.

The West Indian manatee is distributed in riparian and coastal systems from the Western Atlantic, Caribbean Sea, Gulf of Mexico, to the southeast of Brazil^14,15^. The two recognized subspecies of the West Indian manatee—the Florida manatee (*T. m. latirostris*) and the Antillean manatee (*T. m. manatus*)—are listed as threatened by the IUCN^14,16,17^. Florida manatees range from the Florida peninsula to the north of the Gulf of Mexico^18,19^ and migrate seasonally in the winter to warm water refuges^20^. The overall population size of Florida manatees is relatively well estimated (6350 manatees, 95% CI: 5310–7390^21^) compared to the less studied Antillean subspecies^22^. The Antillean manatee inhabits shallow and warm waters in the Bahamas, Gulf coast of Mexico, the Greater Antilles, and Atlantic coasts of Central and South America^14^. Their populations are small^23^ and genetic analysis has separated them into three biogeographically distinct populations: (1) Florida, Central America, Antilles, and the Caribbean coast of South America; (2) Mexico, Central America, and the Caribbean coast of South America; and (3) the northeastern coast of South America including Guyana and Brazil^24–27^.

Across their range, Antillean manatee populations vary dramatically in their size and abundance. Belize has the largest population of Antillean manatees (approximately 1000 individuals) due to its richness of suitable habitats^28^. The distribution and population status for other regions is unknown or declining^22^ and connectivity between different Antillean manatee populations is poorly understood. Panama is thought to contain approximately 150 individuals and its population trend is currently unknown^29^. Examination of the genetics of Panamanian manatees determined they cluster with Mexico/Belize manatees^30^, which supports the hypothesis that manatees in Panama originated from the Mexico/Belize populations during historical colonization events and/or recent migrations^24^.

Tagging and photo-identification studies have revealed how far West Indian manatees migrate and potentially mix within their geographic distribution (See Deutsch et al. 2022^31^ for a review). Several individual Florida manatees have been documented migrating to Cuba^32^ and the Caribbean coast of Mexico^33^ by matching images of scarring on these animals in both locations. Radio-tagged Antillean manatees in Mexico sometimes migrated over 100 km from Chetumal Bay, Mexico to Belize^34^ while most of the individuals tagged in Belize remained within 25 km of where they were captured^35^. In Panama, a lone female was tagged in the San San Pond Sak region and remained in this region throughout the study^36^. The authors cautiously suggest that the San San Pond Sak system contains habitat suitable to high site fidelity in manatees. However, more research is needed to see if Panama manatees migrate to neighboring countries. Based on these migratory patterns, it is possible that manatee subpopulations in Florida, Belize, and Panama may have differences in their vocal behavior.

Manatees are known to produce a variety of vocalizations that play a fundamental role in their communication, such as during social interactions and maintaining contact between cows and their calves^37–40^. Compared to other marine mammals, manatees have a relatively small vocal repertoire composed of five or six distinct call types^11,37,41,42^, depending on the author, that have fundamental frequency values between 0.5 and 5.0 kHz and duration between 0.2 and 0.5 s^38,43,44^. These graded call types contain narrow and broadband vocalizations^11,40^ and have been described as squeaks, high squeaks, squeak-squeals, squeals and chirps^11^. Squeaks, high squeaks and squeals are more commonly observed in reports of Florida manatee communication^37,40,45^. Recent studies found the high squeak is a stereotypical call produced by both Florida^45,46^ and Antillean calves^46^. As the animal grows, the hill-shaped contour of the high squeak appears to flatten and become a more linear call^46^. In addition, results showed few differences between calls produced between captive Florida and Antillean calves^46^.

The Florida and the Antillean manatee have similar vocal tract anatomies^47,48^ and cranial morphology^49^ which are directly related to vocal range capabilities^47,48^. Yet, few studies have focused on geographic variability in wild manatee vocalizations^41,44^. Analyses reported similarities and overlapping distributions in frequency and duration parameters measured between Antillean and Florida manatees^41,44^. However, these studies were based on individuals or small groups, and limited to two geographic locations. Studies addressing geographic variability could provide insight if similarities or differences in genetic variation is a factor in vocal variation in manatees. The aim of this study was to test the hypothesis that vocal characteristics of manatee populations exhibit geographic variability. Specifically, we predict divergent vocalizations between Florida and Antillean subspecies while expecting similarity within Antillean populations in accordance with their geographic proximity. To do this, we tested for differences in the acoustic parameters of manatee calls between three different geographic locations which include two subspecies of West Indian manatees (Florida and Antillean) and two regional populations of Antillean manatees.

## Materials and methods

### Sampling and study sites

**Figure 1:**
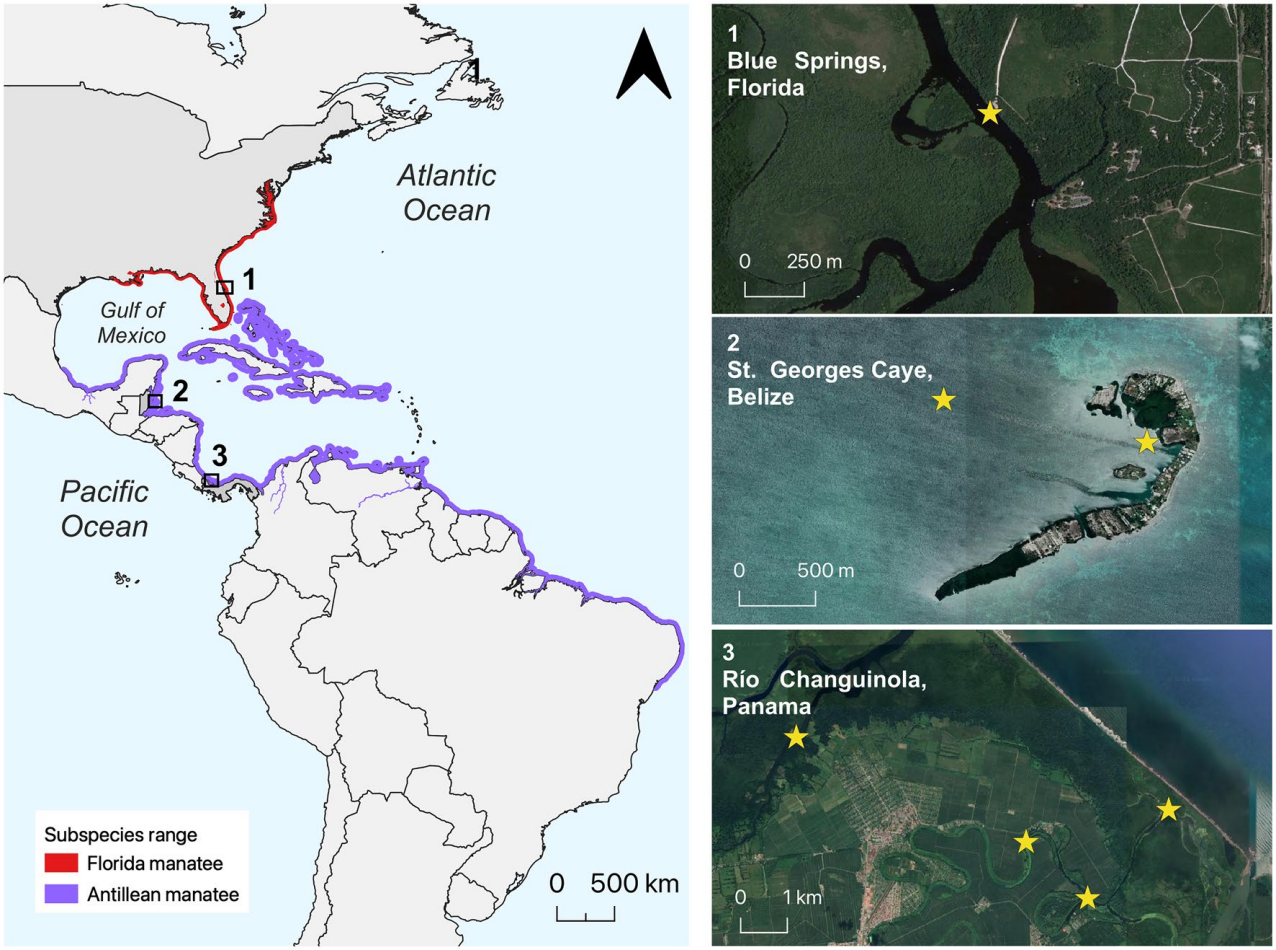
Maps of the Florida and Antillean species range and locations where vocalizations were recorded. Florida manatees (*T. m. latirostris*) were recorded with a drop-in hydrophone at Blue Springs in Florida. In Belize, Antillean manatees (*T. m. manatus*) were recorded with stationary recorders deployed in two different resting holes near St. George’s Caye. Similarly, Antillean manatees in Panama were recorded with stationary recorders deployed in four locations throughout the Río Changuinola. Maps were generated in QGIS v. 3.28.1 using Google Satellite Imagery (URL: https://mt1.google.com/vt/lyrs=s&x={x}&y={y}&z={z}).

Wild manatees were recorded in Florida, Belize, and Panama (Fig. 1). Florida manatees were recorded at Blue Springs, Florida (hereafter “Florida”), a warm, nearly crystal-clear freshwater refuge utilized by manatees when ambient water temperature drops below 20 °C in the winter. Recordings were made on 03 and 04 January 2010 and were obtained using an omnidirectional SQ26-08 hydrophone (linear frequency range: 0.02–50 kHz; sensitivity: − 169 dB re 1 V/µPa, 48 kHz) attached to a M–Audio MicroTrack 24/96 recorder (48 kHz sample rate; 16 bit). The hydrophone was suspended at a depth of 1 m below the surface in water that was 1.5 m deep.

Vocalizations of Antillean manatees in Belize were recorded at St. George’s Caye, a small crescent-shaped island located 9.5 km east of mainland Belize and 2.5 km west of the Belize Barrier Reef (hereafter “Belize”). The site is surrounded by expansive seagrass flats and sand patches with an average depth of 2 m, deep channels (2–3 m deep), and deep depressions (2–4 m) used by manatees as resting holes (i.e., depressions of high use for Belizean manatees^50^). Outside of the deep channels and resting holes, water visibility is clear from the surface to the seabed. The area is regularly inhabited by manatees of all ages and sex classes^51^. Recordings were made during one week in July 2017 and 2.5 weeks in January 2018 using a SoundTrap 300 HF (Ocean Instruments, New Zealand). The recorder sampled at a frequency of 288 kHz (16-bit, flat frequency response: 0.02–150 kHz ± 2 dB, clip level: 172 dB re: 1 µPa) with the preamplifier gain on. The device was anchored to the seafloor in a seagrass bed adjacent to a resting hole with a cinderblock and suspended in the water column at a depth of 1 m above the seafloor in water 1.5 m deep.

In Panama, Antillean manatees were recorded in the Changuinola River (sites S2 and S4, see more information in Merchan et al., 2019^52^), which consists of sinuous, narrow (< 20 m) brackish channels with low visibility and abundant surface and subaquatic vegetation (hereafter “Panama”). Recordings were made from April to May each year from 2015 to 2018 using Wildlife Acoustics Song Meter SM3 Marine bioacoustics programmable hydrophones (Maynard, MA, USA). Hydrophones were installed on PVC placed 1 m above the river floor at 2–3 m depth. The recorder was set up to record 2 min clips every 10 min with sampling frequency set at 96 kHz (32 bits), with the gain set at 35 dB and the sensitivity at 12 dB re: 1 V/µPa (frequency response: 0.02–192 kHz ± 5 dB; dynamic range: 81–165 db SPL).

The numbers of manatees during recording periods at each location was estimated. In Florida, manatees were counted (*n* = 120 animals) during a visual survey at the beginning of the recording session. The number of wild Antillean manatees in recordings from Belize ranged from 17–60 individuals, which were observed during drone flights at St. George’s Caye from 2017 to 2018^53,54^. Between 1 and 15 manatees were present at a time in the recording area. Wild manatees in Panama were estimated at about 45–48 individuals, according to the vocalization clustering method used in Merchan et al. (2019)^52^.

Recording procedures for Florida manatees were approved by the US Fish and Wildlife Service LOA #63658B. Data collection in Belize was conducted under permits granted to E.A.R. from the Belize Fisheries Department (Ref. No. 000031-17, 000010-15). For Panama, the Government of Panama and the Ministerio de Ambiente provided research permits for accessing the river and the protected area. The Animal Care and Use Committee of the Smithsonian Tropical Research Institute (STRI) approved all procedures used in the work. All methods were performed in accordance with relevant guidelines and regulations as suggested in the ARRIVE guidelines.

### Acoustic call selection

Analysis included a total of 15 h recordings from Florida, 116 h from Belize, and 400 h from Panama. Recordings from Florida and Belize were visually inspected in spectrograms, and all calls were manually selected using Raven 1.5 software^55^. Calls from Panama were obtained through automated detection (for further details see Merchan et al. 2019^52^). This resulted in an initial pool of 11,328 vocalizations from Florida, 3262 vocalizations from Belize, and 4819 vocalizations from Panama. Underwater sounds produced close to the surface are subject to the physical limitations of the Lloyd Mirror Effect^37^. Interference patterns can result in a loss or attenuation of lower frequencies^56,57^ which may result in the loss of the fundamental frequency and the second harmonic being incorrectly distinguished as the fundamental. For this reason, we measured the harmonic interval of calls to ensure they corresponded with the measured fundamental frequency, and excluded calls that did not have a fundamental frequency. From this set, non-overlapping calls with a signal-to-noise ratio (SNR) ≥ 6 dB, with clear and identifiable parameters were selected. This left us with a pool of 1,691 vocalizations from Florida, 377 vocalizations from Belize, and 810 vocalizations from Panama for further analysis.

**Figure 2:**
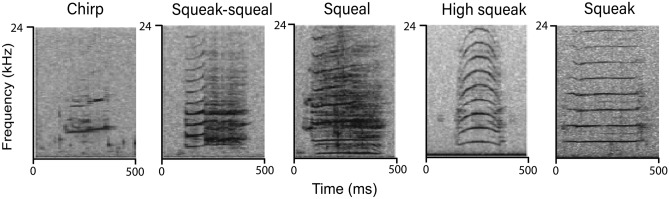
Spectrograms of representative samples of visually classified call types as defined by Brady et al. 2020^42^.

Remaining calls were visually categorized into five categories: squeaks, high squeaks, squeals, squeak-squeals and chirps based on the classification scheme of Brady et al. 2020^11^ (Fig. 2).

Squeaks and high squeaks were differentiated based on the shape of the call contour. Squeals had deterministic chaos throughout the call. Squeak-squeals contained elements of squeaks and squeals and chirps contained frequency jumps. Calls were visually classified by two independent reviewers. If they disagreed on classification, a third skilled reviewer made the final decision of call type. Classification of call types by location is in Table 1.

**Table 1:** Summary of number of visually classified calls, from each geographic location.

Location	Squeak	High squeak	Squeal	Squeak- squeal	Chirp
Belize	203	87	87	0	0
Panama	718	81	7	0	4
Florida	977	455	185	17	57

To be comparable with previous studies on measured characteristics of West Indian manatee vocalizations^11,41,43,46^, six parameters were characterized from the fundamental frequency (the first harmonic) of each vocalization: *Minimum and Maximum Frequency (Hz)* (measured from the power spectrum with a 10 dB threshold); *Duration (s)* (measured from the waveform); *Peak Frequency (Hz)* (frequency at which peak power occurs within the selection^58^); *Bandwidth (Hz)* (value of the maximum frequency minus the minimum frequency); and *Center Frequency (Hz)* (the frequency that divides the sound into two frequency intervals of equal energy^58^). Minimum and maximum frequency were calculated by generating the average power spectrum of the entire measurement box around each call with a 10 dB threshold: minimum was the point of intersection on the left of the plots and maximum was the point of intersection on the right (see Fig. 1 in Brady et al.^46^). Spectrograms for recordings from Florida were calculated with a time resolution of 5.33 ms and a frequency resolution of 46.8 Hz (DFT: 1024; Hanning window; 50% overlap). Belize and Panama recordings were downsampled to 48 kHz, then calculated with a time resolution of 5.33 ms and a frequency resolution of 46.8 Hz (DFT: 1024; Hanning window; 50% overlap).

### Statistical analysis

#### PERMANOVA and SIMPER analysis

Given the results of the classification analyses, our statistical analysis focused on comparing call types with sufficient sample sizes in more than one location. Squeaks and high squeaks were compared across all three locations and squeals were compared between Belize and Florida. To identify differences in call types across populations (Florida, Belize, and Panama), the permutational analysis of variance (PERMANOVA) was run. PERMANOVA is a non-parametric, multivariate test commonly used to compare groups^59,60^. PERMANOVA has been applied in comparisons of vocal characteristics between groups of dolphins^61^ and dolphin vocalizations in different locations^62^. Since one animal may have contributed more than one call to the sample in each location, we ran a repeated measures PERMANOVA. To have equal sample sizes within each call category, calls were randomly selected from larger sample sizes to equal the lowest sample size in the group. For example, we randomly selected 203 squeaks from Florida and 203 squeaks from Panama to equal the lowest sample size of squeaks from Belize (*N* = 203). For high squeaks, we measured 81 calls from each location and 87 squeals were measured from Belize and Florida. The PERMANOVA assumption of homogeneity was assessed for each call category. The only call category that violated the assumption was squeals. However, PERMANOVA is robust to heterogeneity for balanced designs (i.e. equal sample size)^63^ so we continued the analysis with squeals. For each call category (squeaks, high squeaks and squeals), the PERMANOVA was performed with 999 permutations with a Bray–Curtis dissimilarity. A Dunn’s post hoc for each measured variable was conducted for groups that showed significant differences from the results of the PERMANOVA. Lastly, after z transforming the data, we applied a SIMPER analysis technique, which provides the percentage contribution of each variable that differentiates between groups^64^. Statistical analyses were performed using the software PAST 4.12^65^.

#### Stepwise discriminant function analysis

In addition to the SIMPER analysis, stepwise discriminant function analyses (SDFA) were used to determine which parameters were the most important in determining differences between geographic locations^66^. Although most frequency variables were highly correlated (Supplementary Table 1), all variables were retained to minimize loss of information^67^. A leave-one-out cross-validation discriminant analysis was used to predict how the model would perform on new observations. Prior probabilities were obtained from the number of vocalizations used in analysis for each call type. Statistical analyses were performed in SPSS version 27^68^.

## Results

**Figure 3:**
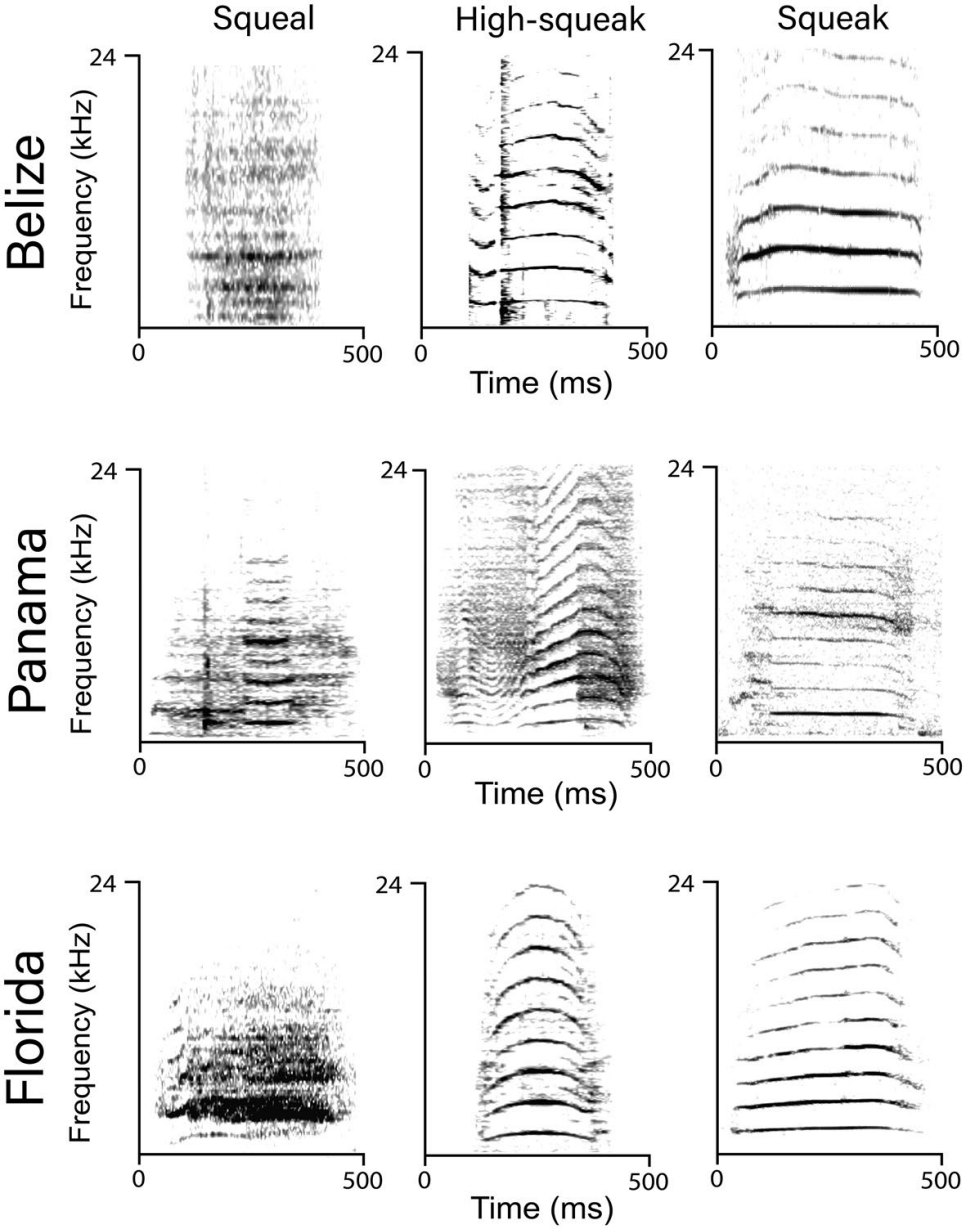
Spectrograms of the vocalizations of the two subspecies of the West Indian manatee recorded in Belize, Panama, and Florida. Recordings of the Antillean manatee were made in Belize and Panama and recordings of the Florida manatee were gathered in Florida. Spectrogram parameters: DFT: 1024; Hanning window; 50% overlap; time resolution: 5.33 ms; frequency resolution: 46.8 Hz.

**Figure 4:**
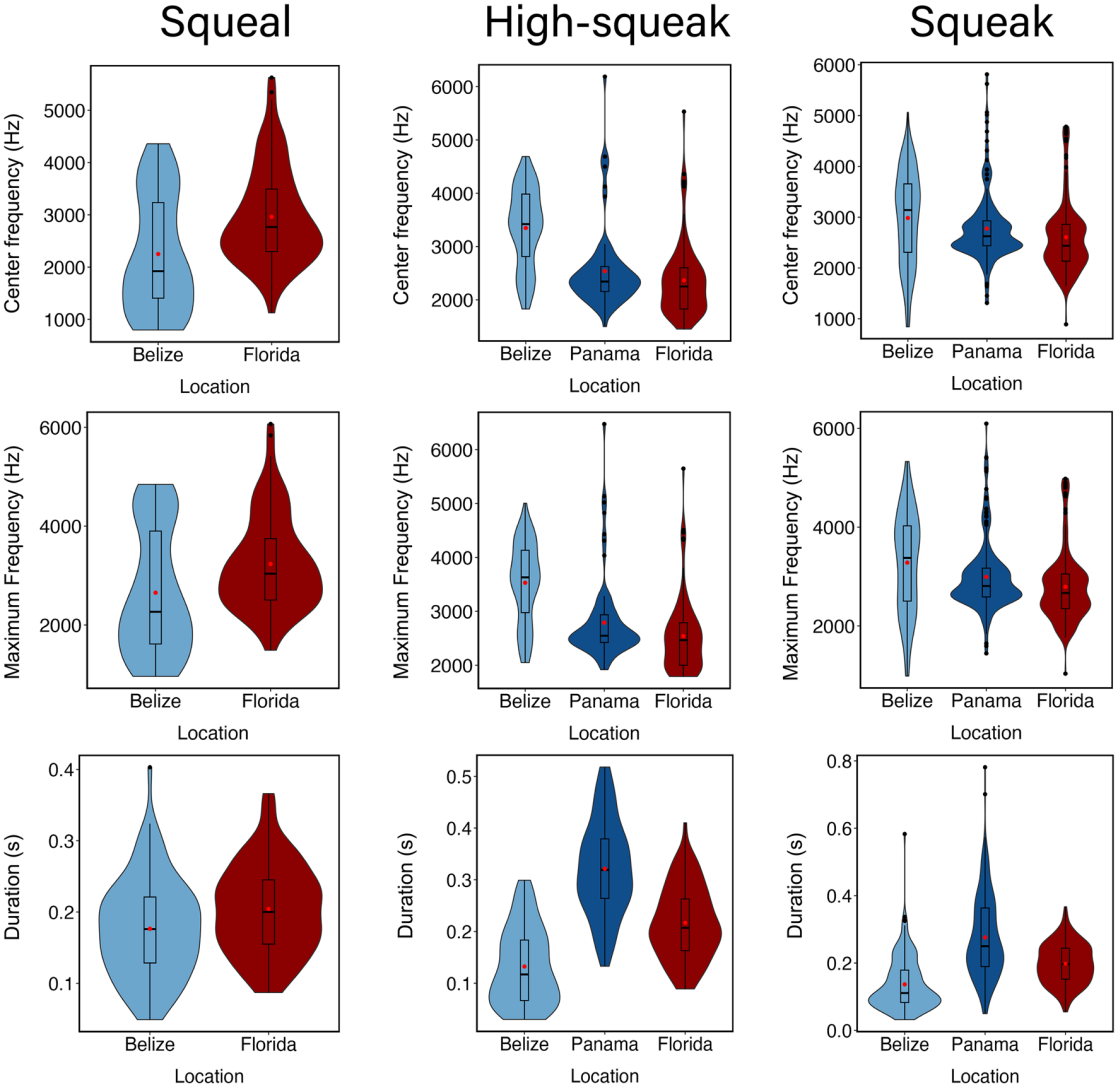
Violin plots and boxplots illustrating the values of three different acoustic parameters (center frequency, maximum frequency, and duration), compared across the three geographically distinct manatee populations. The boxplot represents the upper and lower quartile values. The black line represents the median and the red dot represents the mean. Plots for Antillean manatees are in blue and Florida manatees in red.

All vocalizations recorded from the three populations shared fundamental frequency ranges between 0.5–5.0 kHz and 0.05–0.6 s in duration. Representative samples of calls from each of the three populations are shown in Fig. 3. Means and standard deviation for all variables measured for squeaks, high squeaks, and squeals are in Supplementary Table 2. Violin plots for duration, center and peak frequency were generated in R using ggplot2^69^ and are shown in Fig. 4.

### PERMANOVA and SIMPER analysis

The repeated measures PERMANOVA analysis indicated there were significant differences between characteristics of high squeaks (Pseudo F _252_ = 43.93) and squeaks (Pseudo F _606_ = 13.73) among the three populations (*p* < 0.05). Dunn's post hoc test (Supplementary Table 3) for squeaks showed significant differences for all measured variables (*p* < 0.05) between each location. For high squeaks, Dunn's post hoc test revealed no significant differences for center frequency (*p* = 0.127), peak frequency (*p* = 0.166) and minimum frequency (*p* = 0.520) between Florida and Panama. Belize squeaks and high squeaks were shorter in duration and higher in frequency than those from Florida and Panama. Panama squeaks and high squeaks were longer in duration than Belize and Florida. For squeals, the PERMANOVA results (Pseudo-F_172_ = 26.75) indicated statistical differences (*p* < 0.01) between Belize and Florida. Dunn’s post hoc test results indicated all measured acoustic parameters of squeals were statistically different (*p* < 0.05) between Belize and Florida. Belize squeals were lower in frequency and shorter in duration than squeals from Florida.

The SIMPER analysis indicated almost all frequency and temporal parameters contributed to the differences observed between and within populations. Duration contributed the most to the dissimilarity between Belize-Panama for squeaks and high squeaks as well as squeaks from Panama-Florida. For high squeaks, bandwidth, maximum and peak frequency contributed the most to the dissimilarity observed between the Florida and Antillean subspecies. Center frequency followed by peak and maximum frequency contributed the most to the dissimilarity observed between Belize and Florida for squeals (Table 2).

**Table 2:** Percentage that each variable contributed to the total dissimilarity observed within and between populations for each call type from the SIMPER analysis.

Call type	Maximum frequency	Minimum frequency	Peak frequency	Center frequency	Bandwidth	Duration
Squeak						
Belize-Florida	14.61 (− 21.33)	24.30 (− 35.48)	18.79 (− 27.44)	17.05 (− 24.90)	13.47 (− 19.67)	11.79 (− 17.21)
Belize-Panama	16.79 (− 126.3)	16.68 (− 125.5)	15.86 (− 119.3)	15.65 (− 117.7)	5.71 (− 43.01)	29.30 (− 220.3)
Panama-Florida	< 1 (2.78)	14.72 (− 11.77)	2.18 (− 1.74)	9.24 (− 7.38)	23.04 (− 18.41)	54.31 (− 43.41)
High squeak						
Belize-Florida	23.78 (4.41)	2.21 (0.41)	19.75 (3.66)	< 1 (0.001)	51.68 (9.58)	2.50 (0.47)
Belize-Panama	20.05 (− 49.75)	19.10 (− 47.40)	24.96 (− 61.94)	< 1(− 0.02)	13.73 (− 34.06)	22.15 (− 54.97)
Panama-Florida	49.10 (9.19)	43.19 (8.09)	46.27 (8.66)	< 1 (0.001)	< 1 (− 5.26)	< 1 (− 1.96)
Squeal						
Belize-Florida	23.64 (− 112.1)	23.16 (− 109.90)	24.45 (− 115.00)	24.91 (− 118.1)	< 1 (5.44)	5.18 (− 24.58)

Dissimilarity values for each parameter are in parentheses.

### Stepwise discriminant function analysis

The results of the SDFA for each call type are provided in Supplementary Tables 4–6, including the standardized canonical coefficients, eigenvalues, wilks lambda, chi-square, degrees of freedom, cumulative variance, and *p*-values. For squeaks and high squeaks, there were two significant discriminant functions (*p* < 0.001). Two parameters described 100% of the variance in squeaks. Duration was the most important in determining differences between geographic locations followed by bandwidth. For high squeaks, three parameters described 100% of the variance which included (in order of importance): center frequency, maximum frequency, and duration. Squeal results indicated one significant discriminant function (*p* < 0.001) and the most important parameter was maximum frequency, followed by minimum frequency and duration. The leave-one-out classification analysis classified 60.9% of squeaks, 73.3% of high squeaks, and 72.4% of squeals to the correct geographic location (Table 3). All correct classification scores were greater than expected by chance (squeaks and high squeaks: 1/3 = 33%; squeals: ½ = 50%).

**Table 3:** Classification matrix displaying percentage of calls correctly classified to geographic locations (bold values) compared to calls incorrectly classified to the wrong location (non-bold) according to the leave-one-out stepwise discriminant analysis for squeaks, high squeaks, and squeals.

	Belize	Florida	Panama
Squeaks (*n* = 203 from each site)			
Belize	**64.0**	25.1	10.8
Florida	10.4	**65.3**	24.3
Panama	20.6	26.0	**53.4**
High squeaks (*n* = 81 from each site)			
Belize	**80.5**	11.5	8.0
Florida	11.5	**71.3**	17.2
Panama	6.2	25.9	**67.9**
Squeals (*n* = 87 from each site)			
Belize	**70.1**	29.9	
Florida	25.3	**74.7**	

## Discussion

Our study revealed statistically significant differences in acoustic parameters of classified call types between the Florida and Antillean subspecies at the geographic scale as well as within subspecies (Belize and Panama). Both the SIMPER analysis and SDFA suggest duration plays a significant role in distinguishing between Antillean populations for squeaks and high squeaks, as well as between Florida and Antillean subspecies for squeaks. Additionally, they concur that maximum frequency is a critical parameter in differentiating between Antillean populations for squeals and between Florida and Antillean subspecies for high squeaks. The variances in parameter importance between the two analyses can be attributed to differences in their methodologies. The SIMPER analysis computes pairwise Bray–Curtis dissimilarity within and between groups^64^ whereas SDFA utilizes a stepwise variable selection procedure to iteratively include or exclude one variable at a time until the optimal discriminant function is achieved^66^. Furthermore, previous research has also suggested differences in vocal parameters between populations of the Antillean subspecies but were not statistically analyzed. For example, Ramos et al*.* (2020)^13^ found that the mean fundamental frequency of calls produced by Antillean manatees in Belize was lower than those reported for other populations from Central and South America, but higher than the calls reported from Puerto Rico. High squeaks are produced by both Antillean and Florida manatee calves, while adults produce squeaks^37,45,46^. This study indicates differences in acoustic parameters between adults (squeaks) and calves (high squeaks) between geographic locations. Alternative explanations of these differences may be due to differences between subspecies, geographic separation, differences in sex, current and/or historical connections between populations, habitat, and the number of manatees recorded.

Differences in the acoustic parameters of manatee vocalizations could be due to geographic separation within and across subspecies, and restricted gene flow between manatee populations^70–72^. Geographic isolation contributes to local adaptation to different environments associated with phenotypic and genetic divergence that can influence vocal characteristics in closely related species^73^. Genetic differences found between the Antillean and Florida manatees support their subspecies classification^19^ and their different biogeographic groupings^24,74^. Variation in vocal characteristics between Florida and Antillean manatees are expected based on geographic distance. Yet, this study observed higher dissimilarity scores, and showed a gradient in certain acoustic parameters for high squeaks and squeaks between Antillean manatee populations. Therefore, geographic distance alone cannot explain the variation observed. One explanation could be related to body size. Body condition analysis of 380 wild caught Antillean manatees from Puerto Rico, Cuba, Mexico, Belize, Colombia, and Brazil revealed two distinct ecotypes that were differentiated based on body size^75^. Further, Florida manatees are generally larger than Antillean manatees^76–78^. Our study showed high squeaks and squeaks from Florida manatees were lower in frequency than Belize and Panama. Differences in body size can influence frequency and temporal parameters^79^ and therefore, may be a source of the variation observed in this study.

Differences in the number of manatees we recorded at each site could explain some of the variation detected in their vocalizations across populations. Recordings in Florida involved substantially more manatees at a single time (*n* = 120 individuals) than recordings gathered over 3.5 weeks in Belize (range: 17–60 individuals^53^) and Panama (range: 45–48 manatees^52^). The number of individuals recorded in the area can influence vocal complexity, which in turn can influence the size of the vocal repertoire or diversity of call types produced^80^. For example, chickadees produced a greater diversity of note types as the number of individuals in the group increased^81^. Brady (2020)^82^ observed 171 aggregations of manatees that ranged in size from 1 to 20 animals. They found the occurrence of call types (specifically chirps and squeak-squeals) increased with the number of animals. In this study, we could only estimate how many individuals were present during the recording time frame and it is unknown if all animals present were vocalizing. Although, it is probable that the fewer number of animals estimated in Panama and Belize influenced the lack of chirps and squeak-squeals recorded from these locations.

Maximum, peak and minimum frequency contributed to the variation between locations for each call type which could also suggest sex differences influence variation in calls. In captive environments, where most studies of manatee communication have been conducted, previous work has shown that temporal and frequency parameters differ with the sex of manatees^42,43^. Several studies have reported that females have higher values of maximum and minimum fundamental frequency, but lower mean peak frequencies with respect to males^43,83^. The unknown distribution of sexes in the populations recorded in this study cannot be ruled out as a source of variation found in the three sites and further research is needed to examine these factors.

Although the characteristics of each of the habitats in this study were not measured, differences between habitats could have influenced the observed variation. Studies of geographic variability in the vocalizations of some marine mammal species suggest that different environmental conditions (e.g., water depth, sediment type, pH, salinity, and temperature) can influence regional variation in vocal parameters^84–86^. While all three recording sites were shallow water habitats, Florida and Belize manatees were recorded from fresh and salt water environments respectively. Animals have also been observed to adjust vocal parameters of their calls due to environmental factors varying within their habitats. For example, Amazon river dolphins (*Inia geoffrensis*) altered the duration, center frequency, maximum frequency, and bandwidth of vocalizations depending on whether they were in clear or rich-sediment water^87^. For this study, almost all the previously mentioned parameters were different between geographic locations for squeaks and high squeaks. Panama’s riverine habitat is different from recording sites in Belize and Florida as it includes four different acoustic subhabitats^88^. Acoustic parameters of vocalizations in Panama may differ as an adaptation to communication in diverse habitats with different propagation characteristics.

Ruling out individual variation in the current study is difficult due to the inability to reliably localize to the calling animal in all locations. Localization to an individual can be difficult to ascertain in marine mammals due to the number of animals vocalizing (e.g., in spinner dolphins [*Stenella longirostris*]^89^), animals vocalizing in close proximity to one another (harbor seals^90^), and an inability to observe the animal being recorded (sperm whales [*Physeter macrocephalus*]^91^ and bearded seals^85^). Even with the inability to localize to individual animals, the previously mentioned studies still observed variation in vocalizations between geographic locations. The data from this study indicates that the quartile ranges for the measured variables are similar across all locations, despite differences in the number of calls or individuals. Standard errors of variables measured from Florida are lower than those in the other sites, despite the larger sample size and population. These findings suggest that other factors, such as the environment, may be playing a role in the variation of call characteristics. Further research is necessary to better understand the factors contributing to these variations.

Identifying how the acoustic characteristics of manatee vocalizations vary geographically is critical to improving our understanding of their activity in different populations and habitats. For example, their detection in large datasets gathered with stationary passive acoustic recorders can provide insights into temporal trends in their occurrence and behavior^92^. The range of frequencies observed in this study are well within the hearing range of manatees^56,93^, which suggests that differences in frequency could still be perceived by conspecifics. Recent studies have observed a hybrid species between Amazonian and Antillean manatees^94^ in which Amazonian vocalizations are higher in frequency than Antillean species^63^. This suggests that vocal differences may not be a barrier to mating, as observed, for example, in sperm whales^95^. Considering that Florida manatees have been more commonly observed in Antillean manatee habitats (e.g.,^32,33^), it is unlikely that variation in vocal characteristics would be a barrier to communication.

## Conclusions

Our study provides novel insights into the differences in vocal characteristics of the West Indian manatee, which may be due to geographic separation. Variability of manatee vocalizations between isolated groups and subspecies highlights the importance of studying their acoustic behavior in different regions. Generalizing our findings to other manatee populations is limited by the ecosystem characteristics. More research efforts are needed to investigate the influence of habitat, addressing one of the main limitations of the present study. Continued research on the variation in acoustic characteristics of manatee vocalizations in Florida and the Western Caribbean will facilitate improved acoustic monitoring of manatees and understanding of how their vocal behavior is shaped by environmental and evolutionary factors.

## Supplementary Information

Supplementary Tables.

## Data Availability

The datasets used and/or analyzed during the current study are available from the corresponding author upon request.
