# Supplementary material for: Vocalizations of wild West Indian manatee vary across subspecies and geographic location
Source: Sci Rep. 2023 Jul 7;13:11028. doi: 10.1038/s41598-023-37882-8 (PMC10328939; doi:10.1038/s41598-023-37882-8)
Supplement: Supplementary file 1 — Supplementary Tables. [file 41598_2023_37882_MOESM1_ESM.docx]

Supplementary table 1. Correlation matrix showing the correlation coefficient by parameter for each call type analyzed

|  | Center Frequency | Duration | Peak Frequency | Minimum Frequency | Maximum Frequency | Bandwidth |
| --- | --- | --- | --- | --- | --- | --- |
| **Squeaks** |  |  |  |  |  |  |
| Center Frequency | 1.0 | 0.017 | 0.991 | 0.986 | 0.983 | 0.244 |
| Duration | 0.017 | 1.000 | 0.006 | 0.019 | 0.018 | 0.003 |
| Peak Frequency | 0.991 | 0.006 | 1.000 | 0.978 | 0.970 | 0.228 |
| Minimum Frequency | 0.986 | 0.019 | 0.978 | 1.000 | 0.950 | 0.099 |
| Maximum Frequency | 0.983 | 0.018 | 0.970 | 0.950 | 1.000 | 0.458 |
| Bandwidth | 0.244 | 0.003 | 0.228 | 0.099 | 0.405 | 1.000 |
| **High Squeaks** |  |  |  |  |  |  |
| Center Frequency | 1.0 | 0.0136 | 0.984 | 0.967 | 0.989 | 0.212 |
| Duration | 0.136 | 1.000 | 0.152 | 0.160 | 0.128 | .082 |
| Peak Frequency | 0.984 | 0.152 | 1.000 | 0.964 | 0.967 | 0.148 |
| Minimum Frequency | 0.989 | 0.128 | 0.967 | 1.000 | 0.953 | 0.014 |
| Maximum Frequency | 0.989 | 0.128 | 0.967 | 0.953 | 1.000 | 0.291 |
| Bandwidth | 0.212 | 0.082 | 0.149 | 0.014 | 0.291 | 1.000 |
| **Squeals** |  |  |  |  |  |  |
| Center Frequency | 1.0 | 0.048 | 0.990 | 0.978 | 0.979 | 0.327 |
| Duration | 0.048 | 1.000 | 0.050 | 0.000 | 0.084 | 0.220 |
| Peak Frequency | 0.990 | 0.050 | 1.000 | 0.960 | 0.974 | 0.357 |
| Minimum Frequency | 0.978 | 0.000 | 0.960 | 1.000 | 0.925 | 0.139 |
| Maximum Frequency | 0.979 | 0.084 | 0.974 | 0.925 | 1.000 | 0.504 |
| Bandwidth | 0.327 | 0.220 | 0.357 | 0.139 | 0.504 | 1.000 |

Supplementary Table 2. Means and standard deviations of acoustic parameters for squeaks, high squeaks, and squeals by geographic location.

| Call Type and Location | Center Frequency (Hz) | Duration (s) | Peak Frequency (Hz) | Minimum Frequency (Hz) | Maximum Frequency (Hz) | Bandwidth (Hz) |
| --- | --- | --- | --- | --- | --- | --- |
| Squeak (N=203 from each site) | | | | | | |
| Belize | 2984± 914 | 0.136±0.07 | 2983± 918 | 2690±866 | 3281±1008 | 591±376 |
| Panama | 2776±670 | 0.275±0.008 | 2763±698 | 2537±659 | 2992±697 | 454±183 |
| Florida | 2606±701 | 0.198±0.004 | 2605±705 | 2433±709 | 2792±705 | 359±149 |
| High squeak (N=81 from each site) | | | | | | |
| Belize | 3366±714 | 0.136±0.073 | 3347±760 | 3056±718 | 3549±719 | 493±284 |
| Florida | 2379±707 | 0.216±0.068 | 2356±715 | 2189±698 | 2552±709 | 362±119 |
| Panama | 2539±751 | 0.321±0.090 | 2495±777 | 2243±669 | 2789±748 | 546±233 |
|  |  |  |  |  |  |  |
| Squeal (N=87 from each site) | | | | | | |
| Belize | 2249±1099 | 0.176±0.068 | 2280±1120 | 1894±1021 | 2653±1251 | 758±531 |
| Florida | 2962±909 | 0.204±0.062 | 2950±916 | 2708±902 | 3233±935 | 524±275 |

Supplementary Table 3. PERMANOVA and Dunn’s post hoc test p values by call type and measured variable

| Location 1 | Location 2 | Center Frequency | Minimum Frequency | Maximum Frequency | Peak Frequency | Bandwidth | Duration |  |
| --- | --- | --- | --- | --- | --- | --- | --- | --- |
| Squeak: PERMANOVA (Pseudo F _606_= 13.73; p <0.05) | | | | | | | | |
| Belize | Florida | <0.001 | <0.001 | <0.001 | <0.001 | <0.001 | <0.001 |  |
| Belize | Panama | 0.007 | <0.001 | 0.004 | 0.003 | 0.004 | <0.001 |  |
| Florida | Panama | <0.001 | <0.001 | <0.001 | 0.013 | <0.001 | <0.001 |  |
|  |  |  |  |  |  |  |  |  |
| High Squeak: PERMANOVA (Pseudo F _252_= 43.93; p <0.05) | | | | | | | | |
| Belize | Florida | <0.001 | <0.001 | <0.001 | <0.001 | 0.001 | <0.001 |  |
| Belize | Panama | <0.001 | <0.001 | <0.001 | <0.001 | 0.009 | <0.001 |  |
| Florida | Panama | 0.127 | 0.520 | 0.021 | 0.166 | <0.001 | <0.001 |  |
|  |  |  |  |  |  |  |  |  |
| Squeal: PERMANOVA (Pseudo F _172_= 26.75; p <0.01) | | | | | | | | |
| Belize | Florida | <0.001 | <0.001 | <0.001 | <0.001 | <0.001 | <0.001 |  |

Supplementary Table 4. Stepwise discriminant analysis results for squeaks which include standardized canonical coefficients, eigenvalues, Wilks lambda, chi-squared, degrees of freedom, cumulative variance, and *p*-values.

| Variables | Function 1 | Function 2 |
| --- | --- | --- |
| Duration | 0.930 | 0.367 |
| Bandwidth | -0.369 | 0.929 |
| Eigenvalues | 0.459 | 0.088 |
| Cumulative proportion of  variance | 83.9% | 100% |
| Wilks lambda | 0.630 | 0.919 |
| X^2^ | 279.616 | 51.075 |
| Degrees of freedom | 4 | 1 |
| P value | <0.001 | <0.001 |

Supplementary Table 5. Stepwise discriminant analysis results for high squeaks which include standardized canonical coefficients, eigenvalues, Wilks lambda, chi-squared, degrees of freedom, cumulative variance, and *p*-values.

| Variables | Function 1 | Function 2 |
| --- | --- | --- |
| Center Frequency | -1.537 | -2.434 |
| Maximum Frequency | 1.222 | 3.326 |
| Duration | 0.877 | 0.301 |
| Eigenvalues | 1.120 | 0.201 |
| Cumulative proportion of  variance | 84.9% | 100% |
| Wilks lambda | 0.391 | 0.832 |
| X^2^ | 235.509 | 46.015 |
| Degrees of freedom | 6 | 2 |
| P value | <0.001 | <0.001 |

Supplementary Table 6. Stepwise discriminant analysis results for squeals which include standardized canonical coefficients, eigenvalues, Wilks lambda, chi-squared, degrees of freedom, variance, and *p*-value.

| Variables | Function 1 |
| --- | --- |
| Maximum Frequency | 2.290 |
| Minimum Frequency | -1.734 |
| Duration | 0.490 |
| Eigenvalue | 0.383 |
| Cumulative proportion of  variance | 100% |
| Wilks lambda | 0.723 |
| X^2^ | 55.329 |
| Degrees of freedom | 3 |
| P value | <0.001 |
